# KwaZulu–Natal's Successful Fight against Malaria

**DOI:** 10.1371/journal.pmed.0020371

**Published:** 2005-10-04

The resurgence of malaria remains a major global concern. Artemisinin-based drugs are increasingly seen as one of the best hopes for, at last, making progress in the battle against malaria. Trials of artemisinin-based combination therapy (ACT) in control programs in Southeast Asia have been very encouraging. However, we need to know whether similar levels of effectiveness are achievable in Africa, where the majority of the world's cases of malaria are found.

One part of Africa that has seen increases both in the number of malaria cases and in drug resistance is South Africa's KwaZulu–Natal province. The rise in malaria in this area has been dramatic, with a 15–fold increase in cases taking place during the 1990s. Control efforts during this period involved mosquito control with pyrethroid insecticides (which had replaced DDT) and sulfadoxine-pyrimethamine (SP) as a first-line treatment. (SP was introduced in 1988 in response to high levels of chloroquine resistance.)

In the year 2000, new measures were put in place to address KwaZulu–Natal's malaria crisis. The key elements of this new strategy were the introduction of an ACT drug, artemether-lumefantrine (AL), and an intensification of mosquito control efforts. While pyrethroids were retained for indoor residual spraying of western-style structures, DDT was also reintroduced for spraying traditional homesteads. Karen Barnes and colleagues now present the first comprehensive description and evaluation of the program.

The researchers reviewed four years of malaria morbidity and mortality data at four representative health-care facilities within KwaZulu–Natal's malaria-endemic area. They found that, in the year following improved vector control and implementation of AL treatment, malaria-related admissions and deaths declined by 89%, and outpatient visits decreased by 85%. By 2003, malaria-related outpatient cases and admissions had fallen by 99%, and malaria-related deaths had decreased by 97%. There was a marked and sustained decline in malaria throughout the province. AL cured 99% of those study patients who were followed up for 42 days. Consistent with the findings of focus group discussions, a household survey found that self-reported adherence to the six-dose AL regimen was 96%. Two surveys in subsets of patients receiving AL revealed no serious adverse events resulting from the treatment.

These are impressive results, but they are not solely due to the introduction of ACT. As the authors say, “the ready access to treatment in a relatively well-developed rural primary health-care infrastructure, coupled with an effective vector control programme [are] important factors for deriving the greatest benefit from ACT implementation. Equally important are the strong community perceptions that malaria diagnosis and treatment should be sought urgently at public health-care facilities and treatment then completed.” [Fig pmed-0020371-g001]

**Figure pmed-0020371-g001:**
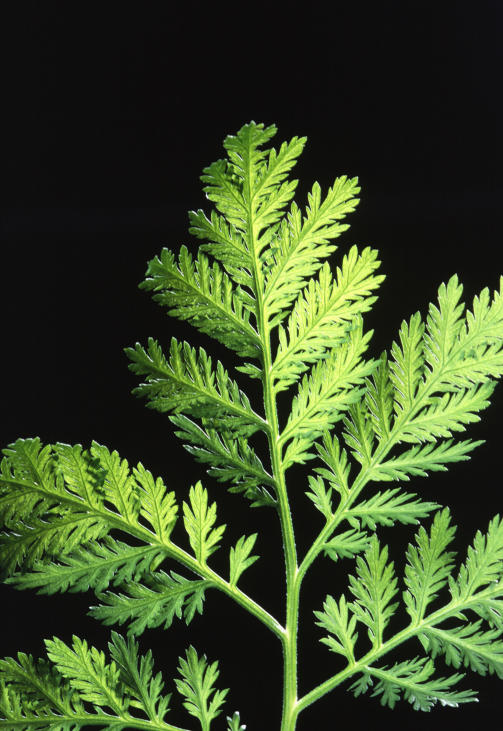
Artemisinin is extracted from *Artemisia annua*, the annual wormwood (Photo: Scott Bauer)

In an accompanying Perspective (DOI: 10.1371/journal.pmed.0020368), Patrick Duffy and Theonest Mutabingwa highlight lessons learned from the “notable success” in KwaZulu–Natal “amid the dire statistics showing a deadly resurgence of malaria.” They also discuss how economic and noneconomic conditions in other parts of sub-Saharan Africa differ from KwaZulu–Natal in ways that are likely to affect the influence of ACT.

